# Virtual reality: an emerging tool for mental health

**DOI:** 10.47626/2237-6089-2024-0973

**Published:** 2025-09-09

**Authors:** Sergio Machado, Rafael Ferreira-Garcia, Lucio Lage Gonçalves, José Carlos Appolinario, Mauro Giovanni Carta, Antonio E. Nardi

**Affiliations:** 1 Universidade Federal do Rio de Janeiro Instituto de Psiquiatria Laboratório de Realidade Virtual na Saúde Mental Rio de Janeiro RJ Brazil Laboratório de Realidade Virtual na Saúde Mental (RVSM), Instituto de Psiquiatria (IPUB), Universidade Federal do Rio de Janeiro (UFRJ), Rio de Janeiro, RJ, Brazil.; 2 UFRJ IPUB Laboratório do Pânico e Respiração Rio de Janeiro RJ Brazil Laboratório do Pânico e Respiração (LABPR), IPUB, UFRJ, Rio de Janeiro, RJ, Brazil.; 3 Instituto Neurodiversidade Centro de Neurociência Queimados RJ Brazil Centro de Neurociência, Instituto Neurodiversidade, Queimados, RJ, Brazil.; 4 UFRJ IPUB Grupo de Obesidade e Transtornos Alimentares Rio de Janeiro RJ Brazil Grupo de Obesidade e Transtornos Alimentares (GOTA), IPUB, UFRJ, Rio de Janeiro, RJ, Brazil.; 5 University of Cagliari Department of Medical Sciences and Public Health Cagliari Italy Department of Medical Sciences and Public Health, University of Cagliari, Cagliari, Italy.

In recent years, the availability and quality of virtual reality (VR) technology have significantly increased.^1^ This has accelerated research on the use of VR in psychiatric settings. Notably, one of the key advantages of VR technology is its ability to create virtual environments that trigger the same feelings, thoughts, and physiological reactions as a corresponding real-life situation would.^2^ The initial application of VR to address mental health began within the context of anxiety^3^ and phobias.^4^ However, VR has also shown increasing promise as an effective therapeutic tool for post-traumatic stress disorders, eating disorders, bipolar disorders, and even schizophrenia.^5,6^

Combined with the fact that virtual environments can be completely controlled, this has made VR technology an intriguing research tool.^2^ What makes VR so appealing to the mental health field is its ability to create controlled, personalized virtual environments in which patients can experience specific situations and scenarios in a safe and supervised manner.^7^ This allows mental health professionals to challenge fears, expose patients to triggering stimuli, and practice coping skills in ways that would be very difficult, dangerous, or even impossible in the real world.^8^ This convenience and the ability to repeatedly practice therapeutic exercises have proven to be beneficial for patient engagement and treatment adherence.^9^ For example, Jung et al.^10^ investigated if personalized VRET could induce anxiety in patients with panic disorder and agoraphobia, due to this being the first step for a successful treatment for anxiety patients. Twenty-eight patients with panic disorder and agoraphobia were divided in personalized and control groups, with three sessions of VR exposure (i.e., theater, train, and elevator scenario) and had self-reported anxiety, skin conductance, heart rate and electroencephalography (EEG) measures registered. During and after the sessions were observed higher levels of anxiety in visual analogue scale when comparing the personalized exposure group to the control group, as well as higher heart rates. Also, EEG activity showed widespread increases in alpha waves in frontotemporal areas in the personalized group compared to the control. Then, personalized VRET has the potential for effective behavioral therapy.

Rather than simply replacing traditional therapies, VR emerges as an instrument capable of enriching and amplifying the therapeutic experience.^1,6,7^ By combining VR with established therapeutic approaches, such as Cognitive-Behavioral Therapy (CBT), mental health professionals can create integrated and more effective interventions.^9,11^ For example, in exposure therapy for phobias, VR allows therapists to create gradual and controlled exposure scenarios that would be difficult or impossible to recreate in base reality. This enables patients to confront their fears in a safe and progressive manner, without the risks and limitations inherent to in vivo exposure.^9^ In bipolar disorder, it has shown that VR can be an effective tool for diagnosing and treating deficits in social intelligence, which are often observed in individuals with bipolar disorder, even during euthymic phases of the illness.^12–14^ VR allows for the simulation of complex social interactions, providing a safe environment for patients to practice and improve their social skills.^13,14^ Parra et al.^14^ assessed the feasibility of a cognitive remediation intervention with fully immersive VR as an additional treatment for bipolar disorder in a randomized controlled cross-over clinical study. Experiment lasted three months, crossing between the experimental group with cognitive remediation fully immersive VR recovery-oriented program plus waiting list and the control group with waiting list. Results showed a good acceptability and tolerability of the experimental group compared to control group, showing significant improvement regarding memory, attention and executive functions, as well as depressive symptoms, emotional awareness and biological rhythms. This is particularly relevant because social intelligence involves both cognitive and affective processing, which are essential components in the "mentalization" and understanding of the mental states underlying behavior.^15,16^ An important mechanism in this context is the relationship between the activation of mirror neurons and social intelligence.^17^ In individuals without bipolar disorder, emotional tasks that require cognitive processing activate specific areas of the brain, such as the right anterior cingulate cortex, which does not occur in the same way in individuals with bipolar disorder.^17^ This insight suggests that VR, by facilitating the practice of social skills, may potentially positively influence the activity of mirror neurons and, consequently, improve social intelligence in bipolar patients.

Furthermore, VR can be used to amplify cognitive restructuring exercises, allowing patients to visualize and experience alternative perspectives in an immersive way.^11^ This can help them identify and challenge dysfunctional thoughts in a more tangible and engaging manner.^11^ Another benefit of VR is its ability to capture behavioral and physiological data from patients during therapy sessions.^18,19^ This data can provide valuable insights to professionals, enabling more accurate assessment of patient progress and more effective adjustment of interventions.^18,19^ The purpose of using VR is not to replace in vivo treatments,^1,20^ but rather to provide an integrated approach, combining VR with face-to-face interventions to achieve even more qualified results.^11^ Throughout the therapeutic process, VR can be used to prepare and familiarize patients with basic real-life situations they will face, developing coping strategies and skills even before they are exposed to real stimuli, in addition to reducing anxiety and increasing confidence.^18^ After virtual exposure, patients can discuss their reactions and learnings with the professional, strengthening the understanding and generalization of the acquired skills.^11^ Lastly, artificial intelligence (AI) can be used to create dynamic and personalized virtual environments, continually adjusting to the patient's needs and emotional responses. This allows for a more immersive and therapeutic experience.^21^ For example, AI-controlled virtual characters can interact with patients in a natural and empathetic way, providing feedback, emotional support, and guidance during treatment.^22^

In terms of limitations, a major challenge is the lack of high-quality research. Many studies use weak methodologies, which prevents conclusive evidence on the efficacy of VR for various mental disorders. There is an urgent need for standardization in VR clinical trials, as there are no clear guidelines for such studies.^23^ Furthermore, the clinical implementation of VR is hampered by the lack of research on the feasibility and efficacy of self-administered treatments, as well as the level of clinician involvement required in this process.^23^ It is also important to conduct pilot trials in diverse communities to assess the cultural appropriateness and acceptability of VR-based mental health interventions.^1^ With regard to accessibility, the clinical adoption of VR faces technological, financial and training barriers that need to be identified and overcome to facilitate its integration into mental health services. Ethical and safety issues are also crucial, especially regarding the integration of artificial intelligence (AI) into VR environments and the safety profile of VR use, with a focus on adverse events.^1^ Technical issues related to VR, such as screen size, resolution, field of view, type of device used, immersive system and sense of presence, are critical to improving clinical outcomes and should be carefully considered. Furthermore, the interaction of VR with other services, such as telemedicine, also presents significant potential to improve therapeutic outcomes.^23^ Therefore, it is essential that there is a greater focus on the development and clinical validation of VR, which could significantly improve the quality of clinical research in this area and, consequently, have a positive impact on the field of mental health.

In conclusion, the increasing use of VR in mental health treatments is an exciting and promising development.^1^ As VR continues to evolve and become more accessible, its adoption in the mental health field is expected to grow even further, benefiting patients and professionals worldwide.^1^

## Data Availability

Not applicable.
